# Effects of a Cognitively Enriched Playful Physical Activity Program on Executive Functions and School Readiness in Preschool Children

**DOI:** 10.3390/bs16071162

**Published:** 2026-07-10

**Authors:** Anis Ben Chikha, Özgür Eken, Birgül Arslanoğlu, Rahma Lousaif, Chiraz Goumni, Nejha Bouallegui, Melih Çalışır, Nizar Souissi, Monira I. Aldhahi

**Affiliations:** 1 The Higher Institute of Sport and Physical Education (Ksar Saïd), University of Manouba, Manouba 2010, Tunisia; anis.benchikha@issepks.u-manouba.tn (A.B.C.); rahma.loussaeif@mjs.state.tn (R.L.); chiraz.goumni@issepks.u-manouba.tn (C.G.); nejha.bouallegui@issepks.u-manouba.tn (N.B.); nizar.souissi@issep.uma.tn (N.S.); 2 Physical Activity, Sport and Health, Research Unit (UR18JS01), National Observatory of Sport, Tunis 1003, Tunisia; 3 Department of Physical Education and Sport Teaching, Faculty of Sports Sciences, Inonu University, Malatya 44280, Türkiye; ozgur.eken@inonu.edu.tr; 4 Department of Humanities and Social Sciences, Faculty of Sciences and Letters, Istanbul Technical University, Istanbul 34469, Türkiye; demirkolb@itu.edu.tr; 5 Research Laboratory (LR23JS01) “Sport Performance, Health and Society”, Higher Institute of Sport and Physical Education of Ksar-Said, Manouba University, Tunis 2010, Tunisia; 6 Department of Physical Education and Sport Teaching, Faculty of Sports Sciences, Bitlis Eren University, Bitlis 13000, Türkiye; mcalisir@beu.edu.tr; 7 Department of Rehabilitation Sciences, College of Health and Rehabilitation Sciences, Princess Nourah bint Abdulrahman University, P.O. Box 84428, Riyadh 11671, Saudi Arabia

**Keywords:** cognitively enriched movement, executive functions, preschool education, attention and inhibition, visuospatial learning, school readiness

## Abstract

Executive functions (EFs) are foundational for early learning and school readiness. Interventions that couple motor engagement with explicit cognitive demands may offer an efficient approach to support EFs during preschool years. This exploratory randomized controlled trial examined the preliminary effects of an 8-week Playful Physical Activities (PPA) program on EF and school readiness. Preschool children were randomized to (i) PPA, a structured program integrating executive challenges (response inhibition, working memory, and planning) into movement games, or (ii) an active control receiving the regular physical education curriculum (3 sessions/week, 60 min/session, 8 weeks). Outcomes were assessed before and after the intervention and included Go/No-Go error rate, BVMT-R immediate and delayed recall, RCFT copy score, and school readiness domains (mathematical, linguistic, and social competence). Group × Time effects were tested using mixed ANOVA and summarized with partial eta squared (η^2^p). Between-group post-test differences were quantified using Hedges’ g. PPA was associated with greater pre-to-post improvements relative to control in attentional control/inhibition (Go/No-Go errors: η^2^p = 0.134; g = −1.78) and visuospatial organization/planning (RCFT copy: η^2^p = 0.307; g = 1.36). Visuospatial memory outcomes showed very large interaction effects (BVMT-R immediate η^2^p = 0.937, g = 5.14; delayed η^2^p = 0.854, g = 4.10), but these values are exploratory and should not be emphasized as the primary evidence for cognitive improvement because the BVMT-R was used as a non-normed raw-score index outside its validated age range in this preschool sample. School readiness indicators showed favorable changes across mathematical, linguistic, and social competence (η^2^p = 0.514, 0.385, and 0.337; g = 1.95, 2.05, and 1.31, respectively). Overall, these preliminary findings suggest that cognitively enriched movement may support EF-related performance and school-readiness indicators, but they should not be interpreted as definitive proof of efficacy. Larger prospectively registered trials with balanced groups, age-appropriate outcome measures, and stronger fidelity documentation are needed before firm recommendations can be made.

## 1. Introduction

The early years of childhood constitute a critical period for cognitive, social, and emotional development, with important consequences for children’s academic success and future lives (Black et al., 2017; Sales et al., 2024). Among the key factors in cognitive development, executive functions (EFs)—including working memory, inhibitory control, and cognitive flexibility—play a decisive role in children’s ability to succeed at school (Cortés Pascual et al., 2019). These skills are not only essential for academic achievement but also promote children’s social and emotional adjustment in group contexts (McClelland et al., 2017). Furthermore, executive functions are important predictors of future academic skills, making it essential to examine these relationships from an early age (Ribner et al., 2017). Executive functions are strongly associated with the maturation of the prefrontal cortex and its functional connectivity with subcortical and cerebellar regions (Diamond, 2013). Contemporary neurodevelopmental models suggest that cognitively engaging physical activities may stimulate these networks through increased synaptic plasticity, enhanced cerebral blood flow, and dopaminergic modulation, thereby supporting self-regulation and working memory processes (Best, 2010; Egger et al., 2019). From an embodied cognition perspective, cognitive processes are not isolated from motor experiences but are grounded in sensorimotor interactions with the environment (Wilson, 2002; Barsalou, 2008). Emerging neuroimaging and intervention studies in early childhood suggest that cognitively demanding motor activities are associated with improved functional connectivity within prefrontal networks supporting inhibitory control and working memory (Diamond, 2016). Accordingly, structured physical activities that integrate cognitive demands may provide an ecologically valid context for strengthening executive functions during early childhood.

Recent research has explored the impact of play-based learning programs, which combine fun and learning, on the development of executive functions in young children (Hassinger-Das et al., 2017). Play-based learning provides interactive and engaging experiences, allowing children to develop cognitive and social skills while stimulating intrinsic motivation to learn (Parker et al., 2022). These programs aim to strengthen key skills such as attention management, emotion regulation, and problem-solving, all of which are essential for school readiness (Yogman et al., 2018). Play is a fundamental component of early childhood education, supporting holistic development (Pyle & Danniels, 2017). The benefits of play in fostering creativity, social skills, and cognitive growth are well established (Skene et al., 2022). Although previous research has demonstrated positive effects of play-based learning and physically active interventions on specific cognitive outcomes, several limitations remain. First, many studies have focused on isolated executive function components without concurrently examining broader school readiness domains such as linguistic, mathematical, and social competencies (Diamond, 2013; Egger et al., 2019). Second, intervention designs often lack structured cognitive–motor integration, instead emphasizing either physical activity or academic content in isolation (Best, 2010). Third, evidence from non-Western educational contexts remains limited, restricting the generalizability of current findings. These gaps highlight the need for randomized controlled trials that simultaneously evaluate executive functions and multidimensional school readiness outcomes within culturally diverse preschool settings. Moreover, relatively few studies have employed randomized controlled designs with clearly defined cognitive–motor integration protocols in preschool populations, limiting causal inference and the precise identification of mechanisms underlying observed cognitive gains.

Howard and Vasseleu (2020) demonstrated that executive functions and self-regulation are strong predictors of future academic skills (Howard & Vasseleu, 2020). Moreover, recent evidence suggests that physically active play interventions can significantly enhance these cognitive processes in early childhood (Egger et al., 2019). However, few studies have examined the combined impact of such programs on both executive functions and school readiness, particularly in preschool children in Tunisia. To address this gap, the present study aimed to evaluate the effects of a structured playful physical activity program on executive functions and school readiness in 5-year-old children in Tunisia. Building upon motor–cognitive integration frameworks and embodied learning theories, the present study extends prior research by implementing a structured playful physical activity program that deliberately embeds executive function challenges within gross and fine motor tasks. Unlike interventions that target either cognitive or academic skills independently, this design simultaneously examines executive functions and key school readiness competencies within a single experimental framework. By situating the investigation within the Tunisian preschool context, this study also contributes cross-cultural evidence to a field predominantly informed by Western samples. By experimentally testing a theoretically grounded cognitive–motor framework, the present study provides preliminary empirical evidence on how structured playful movement may support executive control and school readiness, rather than definitive evidence of intervention efficacy. We hypothesized that children participating in the playful physical activity program would show greater improvements in executive functions, mathematical competence, linguistic competence, and social competence compared to their peers following a traditional physical education program. Executive-function outcomes included selective attention/inhibition, visuospatial memory, and planning; however, because the BVMT-R was used outside its normative age range, BVMT-R scores were treated as exploratory/supportive raw-score indicators rather than decisive evidence of cognitive improvement. Secondary outcomes included school readiness domains (mathematical, linguistic, and social competence). This hypothesis is grounded in the assumption that cognitively enriched physical activities enhance executive control processes, which in turn facilitate the acquisition of foundational academic and social competencies. The integration of movement and cognitive demands is expected to promote adaptive self-regulation, attentional control, and goal-directed behavior, thereby supporting children’s readiness for formal schooling (Diamond, 2013; Best, 2010).

## 2. Materials and Methods

### 2.1. Participants

The study involved 48 preschool children (24 boys and 24 girls) aged 5 years (mean age = 5.0 ± 0.3 years; mean height = 110 ± 5 cm; mean weight = 20 ± 3 kg). Participants were recruited from local preschools in Tunisia using a convenience sampling approach. Although convenience sampling was used due to logistical constraints, the participating preschools were considered representative of typical urban educational settings in Tunisia. Socioeconomic status (SES) was determined based on parental education and occupation, with the sample distribution being 25% low SES, 50% middle SES, and 25% high SES. SES classification followed standardized national educational and occupational categories commonly used in Tunisian demographic research. An a priori power analysis was conducted using G*Power software version 3.1.9.7 (Heinrich Heine University Düsseldorf, Düsseldorf, Germany) to estimate the minimum sample size required to detect intervention effects (Faul et al., 2007). Based on an F-test (ANOVA: repeated measures, within–between interaction), with an alpha level (α) of 0.05, statistical power (1−β) of 0.80, and a medium effect size (f = 0.25), a minimum total sample size of 34 participants was recommended. This medium effect size (f = 0.25) was chosen a priori as a conservative, theory-based estimate of a meaningful cognitive–motor training effect rather than a prediction of the very large effects ultimately observed; the implications of this discrepancy for interpreting the present findings are considered in the Discussion. To account for potential attrition or missing data, 48 participants were enrolled. Participants were then allocated to either the experimental group (EG, n = 19) or the control group (CG, n = 29) using a computer-generated simple randomization sequence without blocking or stratification; consequently, group sizes were not identical.

Inclusion criteria were as follows: aged between 55 and 64 months, enrollment in one of the participating preschools, ability to participate in age-appropriate physical activity sessions, absence of physical or intellectual disabilities that could hinder participation, and written informed consent from parents or legal guardians. Exclusion criteria were as follows: diagnosed neurological, psychiatric, or developmental disorders (e.g., ADHD, autism spectrum disorder), uncorrected visual or auditory impairments that could affect test performance, and use of medication known to affect the central nervous system. Withdrawal criteria were as follows: missing more than 20% of intervention sessions (i.e., more than five sessions) and absence during post-intervention testing.

The study procedures were conducted in accordance with the Declaration of Helsinki (World Medical Association, 2025). Ethical approval was obtained from the local research ethics committee at the High Institute of Sports and Physical Education of Ksar-Saïd (approval reference: CPP N 13/2025). The study was conducted and reported in accordance with the CONSORT (Consolidated Standards of Reporting Trials) guidelines for randomized controlled trials. A completed CONSORT 2025 checklist is provided in the Supplementary Materials. The trial was retrospectively registered at ClinicalTrials.gov under the identifier NCT07678294 (Unique Protocol ID: PPA-EF-SR-2025; public record released on 25 June 2026). Because the actual study start date was 7 April 2025 and the final follow-up/completion date was 23 May 2025, registration occurred after completion of participant follow-up. The PACTR application/record ID 41034 was retained as a secondary registry/reference record.

### 2.2. Study Design and Procedure

**Figure 1:**
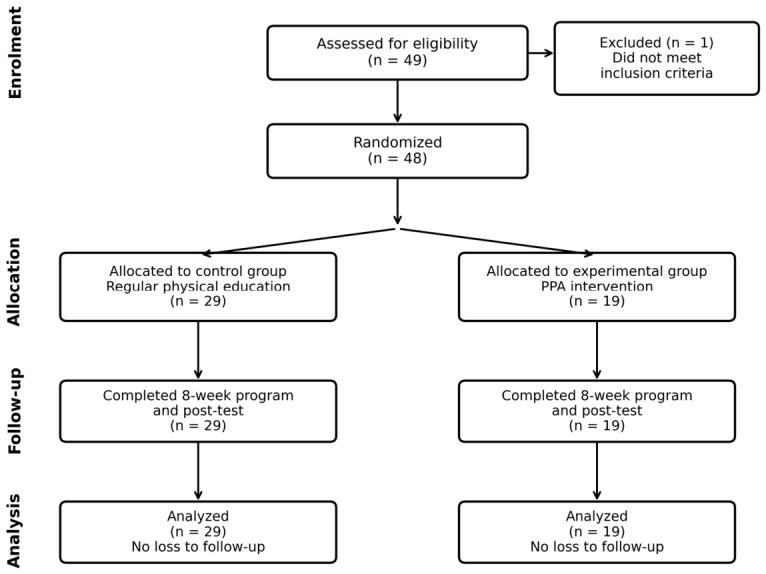
Flow diagram illustrating the experimental procedure design.

The study employed a single-blind, randomized controlled trial (RCT) design with two parallel groups (experimental vs. control). Following enrolment and baseline assessment, participants were allocated to either the Experimental Group (EG) or the Control Group (CG) using a computer-generated randomization sequence. The randomization sequence was generated by a researcher who was not involved in participant recruitment or outcome assessment, and allocation was concealed from the assessors until baseline testing had been completed. To minimize detection bias, group allocation was concealed from outcome assessors, who were independent from the intervention delivery and remained blinded throughout both baseline (pre-test) and post-intervention (post-test) evaluations (Figure 1). Parents, teachers, and children were not blinded due to the nature of the intervention. The intervention lasted eight weeks and consisted of three supervised sessions per week, each lasting 60 min. Both groups followed the same frequency and duration of sessions; however, the EG received the structured Playful Physical Activities (PPA) program, whereas the CG followed a conventional physical education program. All assessments were conducted under standardized conditions at comparable times of day for each child, and identical test instructions and procedures were used at pre- and post-test. A comprehensive battery of tests, described in the Measures section, was administered before and after the intervention to evaluate executive functions, school readiness, and social behavior. The actual study start date was 7 April 2025 and final follow-up/completion occurred on 23 May 2025. No participants withdrew or were lost to follow-up after randomization; all 48 enrolled children completed post-intervention testing and were retained in the analysis (Figure 1).

### 2.3. Intervention Program

Children in the experimental group participated in a structured Playful Physical Activities (PPA) program designed to develop gross and fine motor skills while simultaneously engaging executive functions. The program incorporated point-based games and structured tasks targeting fine motor coordination (e.g., precision activities, hand–eye coordination, and visuospatial manipulation) and gross motor abilities (e.g., running, balance, laterality and coordinated locomotor patterns). A defining feature of the intervention was the deliberate embedding of cognitively demanding elements—namely sustained/selective attention, working memory, inhibitory control, and planning—within movement tasks (i.e., cognitively enriched physical activity). Tasks were delivered in small groups to promote cooperative problem-solving, turn-taking, and rule maintenance, thereby increasing ecological validity for preschool classroom settings. To ensure standardization and intervention fidelity, all sessions were delivered by the same certified instructor trained in the PPA methodology, following a pre-defined session structure (warm-up, main activities, cool-down) and a standardized weekly progression. Representative activities are detailed in Table 1: for example, language-focused sessions used thematic movement games (children moved to images matching a named category before returning to a start line), mathematics-focused sessions embedded counting and sorting into station-to-station locomotor tasks, and social sessions used paired and small-group challenges requiring shared solutions. Difficulty was progressed weekly by increasing the number of rules to be held in mind, the length of action sequences, and the required speed. The planned intervention dose was 24 sessions (3 sessions/week × 8 weeks). Session plans and post-session checklists were used to support standardization and to document completion and deviations; however, the available analytic dataset did not include participant-level attendance, heart-rate/RPE intensity, or independent quantitative fidelity fields. Therefore, attendance, intensity, and checklist-derived fidelity percentages could not be recalculated from the source data and are treated as reporting limitations rather than as estimated post hoc. This absence of participant-level attendance, exercise intensity, and fidelity data prevented dose–response, compliance-threshold, and independent implementation-fidelity analyses. The mapping between specific PPA components and targeted executive function and school readiness domains is summarized in Table 1.

The control group participated in the regular physical education curriculum with the same frequency and duration as the experimental group (three sessions/week, 60 min/session), ensuring an equivalent dose of physical activity exposure. These sessions primarily consisted of free play and traditional age-appropriate physical education activities without systematically embedded cognitive–motor dual-task elements or structured executive function challenges. To reduce contamination, intervention and control sessions were delivered separately and followed distinct session plans aligned with their respective curricula.

### 2.4. Measurements

#### 2.4.1. Inhibitory Control: Go/No-Go Task

Inhibitory control was assessed using a Go/No-Go task, a well-established paradigm for evaluating the ability to suppress prepotent motor responses (Watanabe et al., 2025). Following the procedure described by Dubois et al. (2000), children were instructed to respond (“Go”) as quickly and accurately as possible to designated target stimuli and to withhold responses (“No-Go”) to non-target stimuli (Dubois et al., 2000). Task performance was operationalized as the number of commission errors on the task (i.e., responses incorrectly made to No-Go stimuli), consistent with the error-based metric reported in Table 2 and throughout the Results. Lower scores indicated better inhibitory control. The task was administered under standardized conditions using identical instructions at pre- and post-test.

#### 2.4.2. Visuospatial Memory: Brief Visuospatial Memory Test–Revised (BVMT-R)

Visuospatial memory and learning were assessed using the Brief Visuospatial Memory Test–Revised (BVMT-R; Psychological Assessment Resources, Inc., Lutz, FL, USA) (Benedict, 1997). Children viewed a display of geometric figures for 10 s and were asked to reproduce the figures from memory immediately after stimulus removal (Immediate Recall) and again after a 25–30 min delay (Delayed Recall). The BVMT-R evaluates visuospatial learning, immediate memory, and delayed recall, which are central to non-verbal cognitive processing. Scoring followed standardized BVMT-R guidelines, considering both accuracy and correct placement of reproduced figures; higher scores reflected better performance. The BVMT-R was originally developed and normed for individuals aged 18 years and older; in the present preschool sample, it was therefore used as a non-normed, study-specific index of visuospatial reproduction (raw scores, no published age norms applied). Accordingly, BVMT-R outcomes were interpreted as exploratory/supportive indicators and were not used as the main basis for claims of cognitive improvement. The final analytic dataset contained adjudicated raw scores only and did not retain independent double-scored rater values; consequently, inter-rater reliability could not be recalculated retrospectively. This absence of independent rater-level data further limits the interpretability of the unusually large BVMT-R effects. Observed raw-score ranges in the full sample were 0–20 at baseline and 1–35 at post-test for immediate recall, and 0–9 at baseline and 0–12 at post-test for delayed recall.

#### 2.4.3. Planning and Visuospatial Organization

Planning-related visuospatial organization and constructional strategies were evaluated using the Rey–Osterrieth Complex Figure Test (RCFT) (Osterrieth, 1944; Rey, 1997). Children were instructed to copy a complex geometric figure while the stimulus remained visible. To capture the temporal sequence and organizational strategy of drawing, children used a different colored pencil every 45 s, enabling reconstruction of the drawing process over time. Scoring was conducted according to established RCFT procedures (Osterrieth, 1944). Copy accuracy was quantified by dividing the figure into 18 elements and scoring each element based on location and precision (maximum score = 36): 2 points for a correct element in the correct position; 1 point for a correct element in the wrong position or a distorted element in the correct position; 0.5 points for a distorted element in the wrong position; and 0 points when absent. In addition, organizational strategy (reproduction type) was classified using the hierarchical approach described by Rey (1997), ranging from mature, global construction strategies to fragmented approaches (Rey, 1997). For analysis, the original seven categories were consolidated into five types: Type A (construction on the frame; details within the frame [Levels I–II]), Type B (general outline [Level III]), Type C (juxtaposition of details [Level IV]), Type D (details on a confusing background [Level V]), and Type E (reduction to a familiar outline/scribbling [Levels VI–VII]). Higher copy accuracy scores and more mature organizational types indicated better visuospatial planning/organization. The final analytic dataset contained adjudicated RCFT scores only and did not retain independent double-scored rater values; therefore, inter-rater reliability could not be recalculated retrospectively. The RCFT copy score was therefore considered more developmentally appropriate than the BVMT-R for interpreting visuospatial organization, although the absence of independent rater reliability remains a measurement limitation. Observed RCFT copy-score ranges were 4–22 at baseline and 8–23 at post-test; construction-on-frame scores ranged from 0 to 1 at both time points.

### 2.5. School Readiness Measures

School readiness is a multidimensional construct encompassing several developmental domains, including physical/motor development, social–emotional development, cognition, language/literacy, mathematics, approaches to learning, moral/civic development, and health/well-being. In the present study, school readiness was operationalized through three core domains—mathematical, linguistic, and social competencies—selected because they are consistently identified as robust predictors of early learning and successful transition into formal schooling (Sabol & Pianta, 2012; Williams & Berthelsen, 2017). Domain scores were derived using the procedures described below; higher scores reflected higher competence. The mathematical and linguistic competence outcomes were curriculum-based preschool-readiness indicators used in the participating educational setting. Because the available analytic dataset retained only total scores and did not include item-level responses, test manuals, or formal translation/adaptation records, these two outcomes are interpreted as study-specific curricular indicators rather than internationally normed standardized tests. Observed mathematical-competence scores ranged from 4 to 22 at baseline and from 10 to 25 at post-test; observed linguistic-competence scores ranged from 4 to 24 at baseline and from 3 to 29 at post-test. Internal-consistency reliability could not be recalculated because item-level data were unavailable. Consequently, these school-readiness outcomes should be interpreted as context-specific indicators rather than fully standardized developmental assessments.

#### Social Behavior

Children’s social behavior was assessed using the Social Behavior Evaluation Questionnaire (QECS), validated by Tremblay et al. (1987) and derived from the Preschool Behavior Questionnaire (PBQ) (Tremblay et al., 1987). The QECS is typically completed by teachers and evaluates social competence and behavioral adjustment in early childhood settings. Items cover key domains such as social cooperation, interaction, and autonomy. The QECS yields a total score where higher values indicate better social competence; observed total scores in the present sample ranged from 101 to 242. Because the available analytic dataset retained total scores only, internal-consistency reliability could not be recalculated retrospectively. Because teachers were aware of group allocation, ratings on this teacher-completed measure were not blinded (see the limitations in Section 4). This non-blinded teacher rating introduces possible detection bias for the social-competence outcome. Teachers completed the QECS at pre- and post-test based on their observations of children’s behavior in routine classroom contexts.

### 2.6. Statistical Analyses

All statistical analyses were performed using IBM SPSS Statistics for Windows (version 26.0; IBM Corp., Armonk, NY, USA). Descriptive statistics are presented as mean ± standard deviation (SD). Prior to inferential analyses, distributional assumptions were examined using the Shapiro–Wilk test and visual inspection of histograms and Q–Q plots. Homogeneity of variance was assessed using Levene’s test, and the sphericity assumption was not applicable because the within-subject factor (Time) included only two levels. Baseline equivalence between groups was examined with independent-samples t-tests (Welch correction for unequal variances) for all outcomes.

To evaluate intervention effects, a 2 (Group: Experimental vs. Control) × 2 (Time: Pre-test vs. Post-test) mixed-design analysis of variance (ANOVA) with repeated measures on Time was conducted for each outcome. The primary effect of interest was the Group × Time interaction, indicating differential change between groups across the intervention period. When statistically significant interactions were observed, simple effects were examined using pairwise comparisons (pre–post within each group and between-group differences at post-test), with adjustment for multiple testing using the Bonferroni procedure.

To account for potential baseline differences and unequal group sizes, sensitivity analyses were performed using analysis of covariance (ANCOVA), with post-test values as the dependent variable, Group as the fixed factor, and baseline values as covariates; conclusions were compared with the mixed ANOVA results to assess robustness. Adjusted (ANCOVA) results are reported alongside the primary analyses below. Effect sizes for ANOVA were quantified using partial eta squared (ηp^2^) and interpreted using conventional thresholds. Additionally, standardized mean differences between groups at post-test were calculated using Hedges’ g (small-sample corrected). Effect sizes were interpreted as small (0.2 ≤ g < 0.5), medium (0.5 ≤ g < 0.8), and large (g ≥ 0.8) (Cohen, 2013). The alpha level was set at *p* < 0.05 (two-tailed).

## 3. Results

The repeated-measures ANOVA showed significant Group × Time interactions for most outcomes (Table 2), indicating differential pre-to-post changes between groups. For the Go/No-Go task (errors; lower values indicate better performance), a significant interaction was observed (F(1,46) = 7.11, *p* = 0.011, η^2^p = 0.134), along with main effects of Time (*p* = 0.008, η^2^p = 0.142) and Group (*p* = 0.003, η^2^p = 0.182). Errors decreased from 4.47 ± 4.38 to 1.00 ± 0.88 in the experimental group (Δ = −77.6%) and from 5.07 ± 3.31 to 4.72 ± 2.55 in the control group (Δ = −6.8%), with a large post-test between-group effect (Hedges’ g = −1.78). For BVMT-R Immediate and Delayed Recall, Group × Time interactions were significant (both *p* < 0.001; η^2^p = 0.937 and 0.854, respectively). The experimental group improved from 4.68 ± 4.66 to 28.37 ± 3.09 for immediate recall (Δ = +505.6%) and from 1.58 ± 2.04 to 9.58 ± 1.30 for delayed recall (Δ = +506.7%), whereas the control group showed no change (Δ = 0.0% for both). Post-test between-group effects were large (Hedges’ g = 5.14 and 4.10). These BVMT-R effects are retained for transparency but are interpreted as exploratory because the measure was outside its normative age range and because baseline imbalance and scoring documentation limitations could inflate effect estimates. For the Rey Complex Figure Test, the Copy Score showed a significant interaction (F(1,46) = 20.36, *p* < 0.001, η^2^p = 0.307). Scores increased from 14.05 ± 4.88 to 19.95 ± 1.39 in the experimental group (Δ = +41.9%) and decreased from 16.72 ± 4.12 to 15.76 ± 3.73 in the control group (Δ = −5.8%), with a large post-test effect (Hedges’ g = 1.36). Construction on the Frame showed a borderline interaction (*p* = 0.066, η^2^p = 0.071) and a Time effect (*p* = 0.028, η^2^p = 0.101), with post-test g = 0.66. In school readiness, Group × Time interactions were significant for mathematical, linguistic, and social competence (all *p* < 0.001; η^2^p = 0.514, 0.385, and 0.337, respectively). The experimental group increased from 11.16 ± 3.27 to 20.16 ± 2.87 in mathematics (Δ = +80.7%; g = 1.95), from 15.11 ± 6.09 to 24.42 ± 3.04 in linguistic competence (Δ = +61.6%; g = 2.05), and from 198.42 ± 43.03 to 228.89 ± 12.61 in social competence (Δ = +15.4%; g = 1.31), whereas changes in the control group were small (Δ = −5.1%, +3.9%, and ≈0.0%, respectively). Accordingly, the most interpretable pattern of cognitive change is based on Go/No-Go errors and RCFT copy performance, with school-readiness outcomes providing complementary preliminary evidence.

To contextualize the intervention effects and address the unequal group sizes, baseline (pre-test) equivalence between the experimental and control groups was examined for all outcomes (Table 3). The groups were comparable at baseline on most measures; however, the experimental group scored significantly lower than the control group on mathematical competence (t(45.3) = 3.35, *p* = 0.002, d = −0.93) and BVMT-R immediate recall (t(37.1) = 2.29, *p* = 0.028, d = −0.68), with a comparable trend for the RCFT copy score (*p* = 0.057). These baseline imbalances indicate that simple randomization did not achieve full group equivalence and raise the possibility of regression to the mean for the affected outcomes; they are therefore addressed below using covariate-adjusted analyses (ANCOVA) and are revisited when interpreting the corresponding effect sizes.

To account for these baseline differences and the unequal group sizes, covariate-adjusted comparisons (ANCOVA) were conducted for each outcome, using the post-test score as the dependent variable, group as the fixed factor, and the corresponding baseline score as the covariate. After adjustment for baseline performance, the between-group difference post-test remained statistically significant for every outcome. Adjusted group effects were as follows: Go/No-Go errors, F(1,45) = 36.32, *p* < 0.001, η^2^p = 0.447; BVMT-R immediate recall, F(1,45) = 745.10, *p* < 0.001, η^2^p = 0.943; BVMT-R delayed recall, F(1,45) = 323.25, *p* < 0.001, η^2^p = 0.878; mathematical competence, F(1,45) = 40.97, *p* < 0.001, η^2^p = 0.477; linguistic competence, F(1,45) = 54.32, *p* < 0.001, η^2^p = 0.547; social competence, F(1,45) = 42.89, *p* < 0.001, η^2^p = 0.488; RCFT copy score, F(1,45) = 21.27, *p* < 0.001, η^2^p = 0.321; and RCFT construction on the frame, F(1,45) = 5.05, *p* = 0.030, η^2^p = 0.101. Although the two visuospatial-memory outcomes remained statistically significant after adjustment, the adjusted estimates should be interpreted cautiously because the control group showed no individual-level change between pre- and post-test on either BVMT-R measure and because the BVMT-R was used outside its normative age range. Thus, ANCOVA supports the robustness of the general direction of effects, but it does not remove concerns related to baseline imbalance, unequal group size, regression to the mean, or measurement constraints. Overall, the covariate-adjusted results were consistent with the unadjusted mixed-ANOVA findings, but the estimates should be regarded as preliminary.

**Figure 2:**
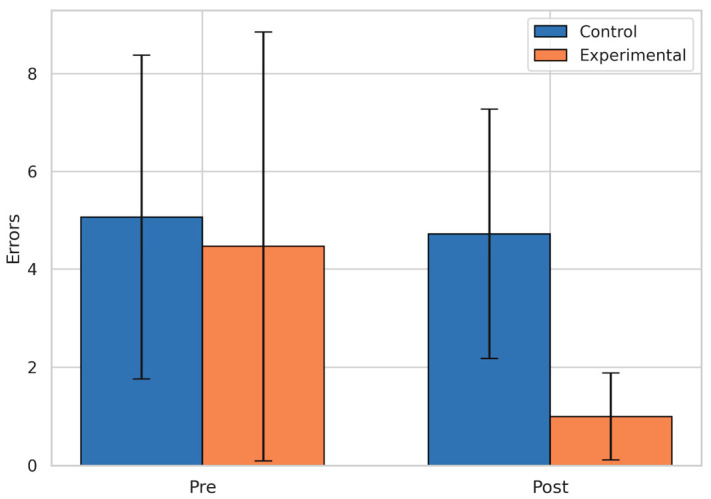
Comparison of selective attention within and between groups before and after the intervention.

A significant main effect of time was observed for selective attention, measured by error rates (F(1,46) = 7.61, *p* = 0.008, η^2^p = 0.142), indicating overall improvements from pre- to post-test. The main effect of the group was also significant (F(1,46) = 10.20, *p* = 0.003, η^2^p = 0.182). Furthermore, a significant time × group interaction was found (F(1,46) = 7.11, *p* = 0.011, η^2^p = 0.134), indicating that improvements in selective attention were predominantly driven by the experimental group. Specifically, the experimental group demonstrated a substantial decrease in errors compared to the control group (Δ = −77.6% vs. −6.8%). The between-group effect size at post-intervention, measured by Hedges’ g, was −1.78, indicating a large effect in favor of the experimental group (Figure 2). Throughout, a negative Hedges’ g for error-based outcomes (Go/No-Go) denotes fewer errors—i.e., better performance—in the experimental group.

**Figure 3:**
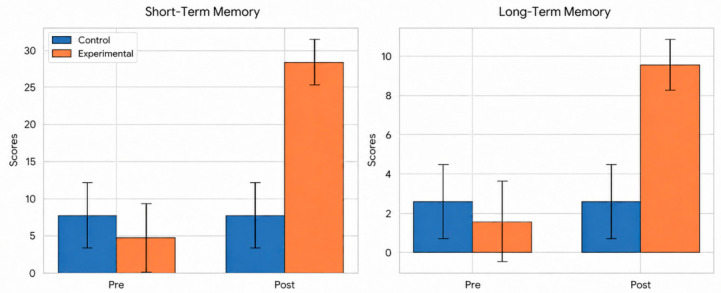
Comparison of short- and long-term memory within and between groups before and after the intervention.

Significant main effects of time were observed for short-term memory (F(1,46) = 451.20, *p* < 0.001, η^2^p = 0.907) and long-term memory (F(1,46) = 175.73, *p* < 0.001, η^2^p = 0.793), indicating improvements from pre- to post-test. The main effect of group was also significant for both short-term memory (F(1,46) = 56.61, *p* < 0.001, η^2^p = 0.552) and long-term memory (F(1,46) = 39.36, *p* < 0.001, η^2^p = 0.461). Furthermore, statistically significant time × group interactions were found for both short-term memory (F(1,46) = 688.67, *p* < 0.001, η^2^p = 0.937) and long-term memory (F(1,46) = 268.21, *p* < 0.001, η^2^p = 0.854). Specifically, the experimental group demonstrated very large increases compared to the control group (short-term memory: Δ = +505.6% vs. 0.0%; long-term memory: Δ = +506.7% vs. 0.0%). Between-group effect sizes at post-intervention, measured by Hedges’ g, were 5.14 for short-term memory and 4.10 for long-term memory, indicating numerically very large between-group differences in favor of the experimental group (Figure 3). These BVMT-R results are reported descriptively as exploratory outcomes only and should not be taken as the principal evidence for intervention-related cognitive improvement. Given the implausible magnitude of these memory effects, the low and significantly lower baseline value for immediate recall in the experimental group, the use of the BVMT-R outside its normative age range, the absence of alternate test forms, the lack of independent rater-level reliability data, and the exact zero-change pattern in the control group, these memory effects should not be interpreted at face value as the magnitude of true intervention-induced learning.

**Figure 4:**
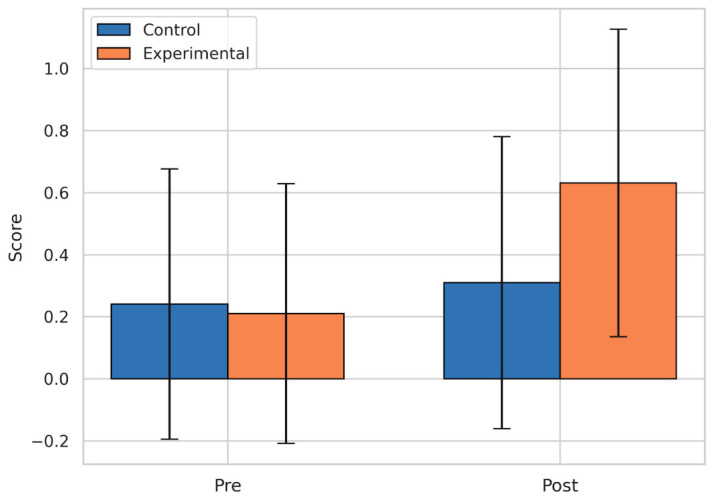
Comparison of planning (construction on the frame) within and between groups before and after the intervention.

A significant main effect of time was observed for planning, specifically construction on the frame (F(1,46) = 5.18, *p* = 0.028, η^2^p = 0.101), indicating improvements from pre- to post-test. The main effect of group was not significant (F(1,46) = 2.26, *p* = 0.140, η^2^p = 0.047). However, a borderline significant time × group interaction was found (F(1,46) = 3.54, *p* = 0.066, η^2^p = 0.071), indicating a trend in which improvements were predominantly driven by the experimental group. Specifically, the experimental group demonstrated a substantial increase compared to the control group (Δ = +200.0% vs. +28.6%). The between-group effect size at post-intervention, measured by Hedges’ g, was 0.66, indicating a medium-to-large effect in favor of the experimental group (Figure 4).

**Figure 5:**
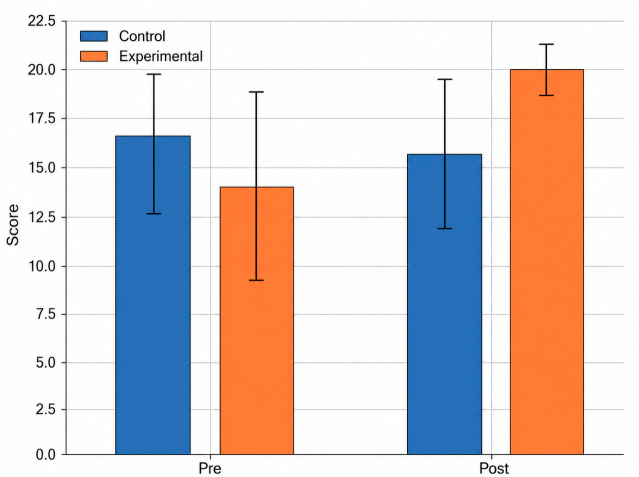
Comparison of planning (score) within and between groups before and after the intervention.

A significant time × group interaction was found for the overall planning score (F(1,46) = 20.36, *p* < 0.001, η^2^p = 0.307), indicating differential change in planning-related RCFT copy performance. Specifically, the experimental group demonstrated a substantial increase in strategic planning and organizational capacities compared to the control group (Δ = +41.9% vs. −5.8%). While the control group’s performance remained broadly stable with a slight decline (from 16.72 to 15.76), the experimental group’s scores increased from 14.05 to 19.95. The between-group effect size at post-intervention, measured by Hedges’ g, was 1.36, indicating a large effect in favor of the experimental group. Because the RCFT copy task is more developmentally interpretable than the BVMT-R in this age group, this outcome is treated as a more credible cognitive indicator, while still acknowledging the absence of independent rater-reliability data. Furthermore, the reduced standard deviation within the experimental group at post-test is consistent with more homogeneous post-test performance in this group (Figure 5).

**Figure 6:**
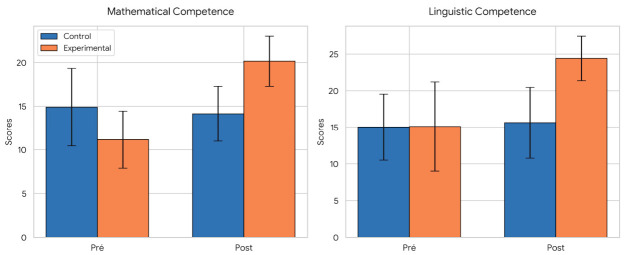
Comparison of school readiness, mathematical and linguistic competence within and between groups before and after the intervention.

Significant main effects of time were observed for mathematical competence (F(1,46) = 20.55, *p* < 0.001, η^2^p = 0.309) and linguistic competence (F(1,46) = 25.78, *p* < 0.001, η^2^p = 0.359), indicating improvements from pre- to post-test. The main effect of group was significant only for linguistic competence (F(1,46) = 15.27, *p* < 0.001, η^2^p = 0.249), while the group effect for mathematical competence was not significant (F(1,46) = 2.10, *p* = 0.154, η^2^p = 0.044). Significant time × group interactions were found for both mathematical competence (F(1,46) = 48.57, *p* < 0.001, η^2^p = 0.514) and linguistic competence (F(1,46) = 28.76, *p* < 0.001, η^2^p = 0.385), indicating that improvements were predominantly driven by the experimental group. Specifically, the experimental group demonstrated substantial increases compared to the control group (mathematical competence: Δ = +80.7% vs. −5.1%; linguistic competence: Δ = +61.6% vs. +3.9%). Between-group effect sizes at post-intervention, measured by Hedges’ g, were 1.95 for mathematical competence and 2.05 for linguistic competence, indicating large effects in favor of the experimental group (Figure 6). Because these measures were curriculum-based and item-level reliability data were unavailable, the findings are interpreted as preliminary school-readiness indicators rather than standardized evidence of broad developmental change.

**Figure 7:**
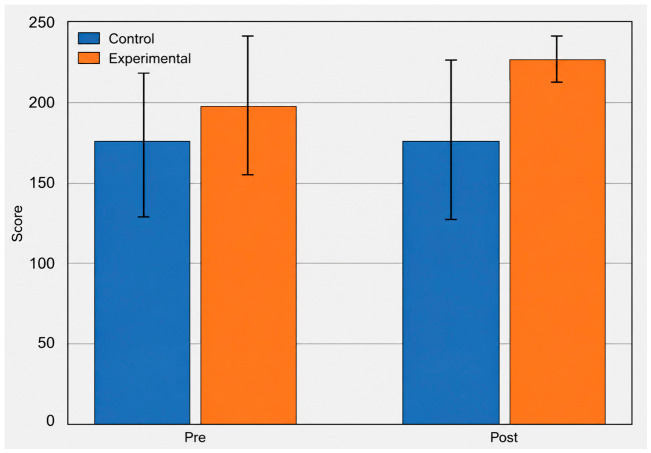
Comparative analysis of changes in social competence levels between the experimental and control groups before and after the intervention. Error bars represent standard deviations.

A significant main effect of time was observed on social competence (F(1,46) = 15.15, *p* < 0.001, η^2^p = 0.248), as well as a significant main effect of group (F(1,46) = 8.95, *p* = 0.004, η^2^p = 0.163), indicating higher overall performance in the experimental group. Furthermore, the time × group interaction was significant (F(1,46) = 23.39, *p* < 0.001, η^2^p = 0.337), reflecting a differential progression between the groups. Specifically, the experimental group demonstrated a substantial improvement (Δ = +15.4%), whereas the control group showed no meaningful change (Δ ≈ 0%). The between-group effect size at post-intervention was large (Hedges’ g = 1.31), consistent with a positive intervention-related difference. However, because social competence was rated by teachers who were not blinded to group allocation, this finding should be interpreted cautiously as potentially susceptible to detection bias (Figure 7).

## 4. Discussion

The present randomized controlled trial examined whether an eight-week Playful Physical Activities (PPA) program—explicitly designed to embed executive-function demands within gross and fine motor tasks—improves executive functions and multidimensional school readiness in Tunisian preschool children. The results provide preliminary, not definitive, evidence that the PPA program was associated with greater pre-to-post gains than the conventional physical education curriculum in selected executive-function and school-readiness outcomes. The most interpretable cognitive effects were observed for Go/No-Go errors and RCFT copy performance, whereas the BVMT-R memory results were treated as exploratory because of the age range and measurement limitations. Overall, the pattern of Group × Time interactions is consistent with the hypothesis that cognitively enriched, structured playful movement may support executive control and early school-readiness skills, but the small and unequal sample and baseline imbalances require cautious interpretation.

Although the numerically largest intervention effects were observed for visuospatial memory (BVMT-R immediate and delayed recall), these outcomes should be interpreted with particular caution and are not emphasized as the main evidence for cognitive benefit. More credible cognitive evidence is provided by the reduction in Go/No-Go errors and the improvement in RCFT copy performance, both of which are more directly interpretable in this preschool sample. These findings align with theoretical and empirical work suggesting that executive functions are malleable during early childhood and can be strengthened by activities that combine physical engagement with sustained attentional control, rule maintenance, response inhibition, and goal-directed sequencing (Diamond, 2013; Best, 2010; Egger et al., 2019). From a neurodevelopmental perspective, cognitively engaging movement may stimulate prefrontal networks implicated in executive control through repeated practice of attention shifting, inhibition, and working-memory updating within motivating, socially meaningful contexts (Diamond, 2016). From an embodied cognition framework, the integration of motor experiences with cognitive challenges offers an ecologically valid learning environment in which children can “practice” executive control as part of action planning and adaptation to task constraints (Wilson, 2002; Barsalou, 2008). These neurodevelopmental and embodied-cognition accounts are interpretive: the present study measured behavioral outcomes only and did not assess neural, physiological, or mechanistic variables. They should therefore be regarded as plausible explanatory frameworks rather than mechanisms demonstrated by these data.

At the same time, the magnitude of several effects—particularly in BVMT-R outcomes—was implausibly large for an 8-week intervention in 5-year-old children. While this may reflect some genuine learning gains induced by a structured cognitive–motor program, alternative explanations should be considered. First, baseline performance in the experimental group was low, creating room for large proportional changes and potentially inflating standardized effect sizes. Second, the BVMT-R was used outside its normative age range in this preschool sample and was interpreted only as a raw-score visuospatial reproduction index. Third, the score distributions suggest possible floor effects at baseline and ceiling compression at post-test, particularly for delayed recall, where observed post-test scores reached the maximum raw value in some children. Fourth, repeated testing can introduce practice effects; although the control group did not show comparable gains in memory outcomes, the exact zero-change pattern in the control group raises concerns about test familiarity, administration, scoring, or data-recording characteristics. Future studies should therefore include additional safeguards such as age-appropriate visuospatial-memory measures, alternate test forms where available, blinded duplicate scoring with reported inter-rater reliability, and pre-registration of primary outcomes with clearly specified scoring procedures. In the present case, the experimental group also started from significantly lower baseline values on these memory outcomes (Table 3), which, together with use of the BVMT-R outside its validated age range and the absence of alternate forms, means the very large standardized effects for visuospatial memory cannot be interpreted at face value as the magnitude of true intervention-induced learning. We therefore treat the BVMT-R findings as exploratory only and do not use them as primary evidence for cognitive improvement. The more conservative cognitive outcomes—Go/No-Go errors and RCFT copy score—provide a more credible basis for interpreting the intervention’s potential cognitive benefit.

Beyond executive functions, the PPA program improved school readiness competencies in mathematics, language, and social functioning. This is notable because many interventions focus on isolated cognitive skills, whereas readiness for formal schooling is multidimensional and includes both academic precursors and social–emotional adaptation (Sabol & Pianta, 2012; Williams & Berthelsen, 2017). The observed gains are consistent with the hypothesis that executive functions act as proximal “learning enablers”: improvements in attention regulation, working memory, and planning can facilitate children’s capacity to follow instructions, sustain goal-directed behavior, and coordinate cognitive resources during early numeracy and language tasks (Cortés Pascual et al., 2019; Ribner et al., 2017). The PPA program likely supported early numeracy through movement-based counting, sorting, and problem-solving games that demand monitoring of rules and outcomes. Similarly, language-related benefits may be explained by repeated opportunities for naming, categorization, verbal instruction following, and peer interaction embedded in structured play—processes known to support vocabulary growth and early literacy precursors (Yogman et al., 2018; Battaglia et al., 2020).

Improvements in social competence also support the role of cooperative, rule-based play in strengthening prosocial behavior, self-regulation, and conflict management. Structured activities conducted in small groups require turn-taking, negotiation, and shared goal pursuit, which can be generalized as classroom social functioning and readiness-related behavioral adjustment (McClelland et al., 2017). Importantly, these social gains complement the executive-function improvements, as self-regulation and social adaptation are tightly coupled during the preschool years.

In contexts where preschool education may be comparatively more academically focused, structured playful physical activities represent a feasible, low-cost pedagogical strategy that can be integrated into daily routines without displacing learning goals. The present results extend the evidence base by providing data from a Tunisian preschool setting, contributing cross-cultural insight to a literature still dominated by Western samples. Embedding cognitive demands within enjoyable movement tasks may be especially valuable in early childhood education because it leverages intrinsic motivation and sustained engagement—two factors that can be difficult to achieve through purely didactic instruction.

This study has several strengths, including a randomized controlled design, assessor blinding, and an intervention structured around explicit cognitive–motor integration. Nevertheless, several limitations should be considered when interpreting the findings. First, the sample was modest, group sizes were unequal because simple randomization was used without blocking or stratification, and baseline differences were present for mathematical competence and BVMT-R immediate recall; ANCOVA reduced but did not eliminate concerns about residual confounding and regression to the mean. Second, the BVMT-R was used outside its normative age range, alternate forms were not used, and independent rater-level scoring data were unavailable; consequently, the unusually large BVMT-R effects are exploratory and should not be treated as definitive evidence of cognitive improvement. Third, implementation documentation was incomplete: participant-level attendance, heart-rate/RPE intensity, and quantitative fidelity percentages were not retained, preventing dose–response, exercise-intensity, compliance-threshold, and independent fidelity analyses. Fourth, independent double-scoring records were unavailable for BVMT-R/RCFT outcomes, item-level reliability data were unavailable for the school-readiness indicators, and social competence was based on non-blinded teacher ratings, creating potential detection bias for that outcome. Fifth, trial registration was retrospective: the study started on 7 April 2025 and final follow-up/completion occurred on 23 May 2025, whereas the ClinicalTrials.gov public record was released on 25 June 2026 under NCT07678294; the PACTR application/record ID 41034 is retained only as a secondary registry/reference record. Finally, the study assessed short-term pre-to-post changes only, and follow-up assessments are needed to determine retention and transfer to later school performance. Together, these limitations mean that the findings should be viewed as preliminary and hypothesis-generating pending replication in larger, prospectively registered, adequately balanced trials with age-appropriate measures and stronger implementation documentation.

## 5. Conclusions

In summary, an eight-week cognitively enriched, structured playful physical activity program was associated with greater gains in selected executive-function and school-readiness indicators relative to conventional physical education in this preschool sample. These results should be interpreted as preliminary evidence rather than definitive proof of intervention efficacy, particularly because the sample was small and unequally allocated, baseline imbalances were present, BVMT-R effects were unusually large and exploratory, fidelity/reliability documentation was incomplete, and trial registration was retrospective. The findings support further investigation of cognitively engaging movement-based plays as a pragmatic approach to strengthening self-regulation, early learning foundations, and social adaptation during the preschool period, but larger prospectively registered trials using balanced allocation, age-appropriate cognitive measures, independent reliability checks, and documented attendance/intensity/fidelity are needed before firm educational recommendations can be made.

## Figures and Tables

**Table 1:** Study protocol for a play-based physical activity program targeting executive functions and school readiness in preschool children.

Phase	Duration	Content	Targeted Functions
Warm-up	7 min	Playful motor games (running, balance, laterality, varied movements) integrating simple rules and attentional cues	Selective attention, inhibition, motor readiness
Main activity	30–35 min	Structured games combining gross and fine motor skills with cognitive demands, organized into three weekly sessions with linguistic, mathematical, and social focus	Executive functions and school readiness
Language skills	1st weekly session	Thematic games (animals, clothing, colors, objects); motor tasks involving movement toward correct images or objects; verbal instruction following, rule memorization, naming tasks; group activities promoting verbal exchanges and turn-taking	Vocabulary enrichment, categorization, working memory, attention, communication, cooperation
Mathematical skills	2nd weekly session	Counting activities integrated into movement-based games; simple calculations; sorting tasks (size, quantity); object manipulation and movement between stations; collective problem-solving	Numeracy, reasoning, logic, visuospatial skills, planning, cognitive flexibility
Social skills	3rd weekly session	Activities in pairs, triads, and small groups; role assignment; collective challenges requiring shared solutions; verbal and non-verbal communication	Social adaptation, responsibility, self-regulation, cooperation, planning
Cool-down	5 min	Calm cooperative games, breathing exercises, verbalization, and collective feedback	Emotional regulation and social communication

**Table 2:** Descriptive and inferential statistics for executive-function and school-readiness outcomes.

**A. Descriptive statistics and percentage change**							
**Outcome**	**Group**		**Pre (M ± SD)**		**Post (M ± SD)**		**Δ (%)**
Selective Attention	Control		5.07 ± 3.31		4.72 ± 2.55		−6.8%
	Experimental		4.47 ± 4.38		1.00 ± 0.88		−77.6%
Short-Term Memory	Control		7.76 ± 4.40		7.76 ± 4.40		0.0%
	Experimental		4.68 ± 4.66		28.37 ± 3.09		+505.6%
Long-Term Memory	Control		2.59 ± 1.88		2.59 ± 1.88		0.0%
	Experimental		1.58 ± 2.04		9.58 ± 1.30		+506.7%
Planning (Construction on Frame)	Control		0.24 ± 0.44		0.31 ± 0.47		+28.6%
	Experimental		0.21 ± 0.42		0.63 ± 0.50		+200.0%
Planning (Score)	Control		16.72 ± 4.12		15.76 ± 3.73		−5.8%
	Experimental		14.05 ± 4.88		19.95 ± 1.39		+41.9%
Mathematical Competence	Control		14.90 ± 4.44		14.14 ± 3.15		−5.1%
	Experimental		11.16 ± 3.27		20.16 ± 2.87		+80.7%
Linguistic Competence	Control		15.03 ± 4.52		15.62 ± 4.84		+3.9%
	Experimental		15.11 ± 6.09		24.42 ± 3.04		+61.6%
Social Competence	Control		177.28 ± 47.97		177.21 ± 48.67		≈0.0%
	Experimental		198.42 ± 43.03		228.89 ± 12.61		+15.4%
**B. Mixed ANOVA effects and post-test between-group effect sizes**							
**Outcome**	***p*** **(Group)**	***p*** **(Time)**	***p*** **(G × T)**	**η^2^p (Group)**	**η^2^p (Time)**	**η^2^p (G × T)**	**Hedges’ g**
Selective Attention	0.003 *	0.008 *	0.011 *	0.182	0.142	0.134	−1.78
Short-Term Memory	<0.001 *	<0.001 *	<0.001 *	0.552	0.907	0.937	5.14
Long-Term Memory	<0.001 *	<0.001 *	<0.001 *	0.461	0.793	0.854	4.10
Planning (Construction on Frame)	0.140	0.028 *	0.066	0.047	0.101	0.071	0.66
Planning (Score)	0.363	0.023 *	<0.001 *	0.018	0.107	0.307	1.36
Mathematical Competence	0.154	<0.001 *	<0.001 *	0.044	0.309	0.514	1.95
Linguistic Competence	<0.001 *	<0.001 *	<0.001 *	0.249	0.359	0.385	2.05
Social Competence	0.004 *	<0.001 *	<0.001 *	0.163	0.248	0.337	1.31

Δ (%) = ((Post mean − Pre mean)/|Pre mean|) × 100 (based on group mean difference). * *p* < 0.05 (two-tailed).

**Table 3:** Baseline (pre-test) comparison between the experimental and control groups.

Outcome (Pre-Test)	Control (n = 29)	Experimental (n = 19)	t	df	*p*	Cohen’s d
Selective attention (errors)	5.07 ± 3.31	4.47 ± 4.38	0.51	31.2	0.617	−0.16
BVMT-R immediate recall	7.76 ± 4.40	4.68 ± 4.66	2.29	37.1	0.028 *	−0.68
BVMT-R delayed recall	2.59 ± 1.88	1.58 ± 2.04	1.73	36.4	0.093	−0.52
RCFT construction on frame	0.24 ± 0.44	0.21 ± 0.42	0.25	39.7	0.807	−0.07
RCFT copy score	16.72 ± 4.12	14.05 ± 4.88	1.97	34.0	0.057	−0.60
Mathematical competence	14.90 ± 4.44	11.16 ± 3.27	3.35	45.3	0.002 *	−0.93
Linguistic competence	15.03 ± 4.52	15.11 ± 6.09	−0.04	30.7	0.966	0.01
Social competence (QECS)	177.28 ± 47.97	198.42 ± 43.03	−1.59	41.5	0.119	0.46

Values are means ± SD computed from the participant-level pre-test data (control n = 29; experimental n = 19). The t, df, and *p* values are from independent-samples tests with Welch’s correction for unequal variances; Cohen’s d is the standardized mean difference, with positive values indicating higher scores in the experimental group. For the Go/No-Go task, scores represent error counts, so lower values indicate better performance. * *p* < 0.05.

## Data Availability

The datasets generated and/or analyzed during the current study are available from the corresponding author on reasonable request.

## Supplementary Materials

The following supporting information can be downloaded at: https://www.mdpi.com/article/10.3390/bs16071162/s1, Table S1: Completed CONSORT 2025 Checklist.
