# Supplementary material for: Effects of a Cognitively Enriched Playful Physical Activity Program on Executive Functions and School Readiness in Preschool Children
Source: Behav Sci (Basel). 2026 Jul 10;16(7):1162. doi: 10.3390/bs16071162 (PMC13405789; doi:10.3390/bs16071162)
Supplement: Supplementary file 1 [file behavsci-16-01162-s001.zip › behavsci-4321536-supplementary.pdf]

Completed CONSORT 2025 Checklist - Revised

MDPI editorial assessment attachment with ethics approval and registration clarification

**Manuscript**	Effects of a Cognitively Enriched Playful Physical Activity Program on Executive Functions and School Readiness in Preschool Children
**Manuscript ID**	behavsci-4321536
**Trial registration**	ClinicalTrials.gov: NCT07678294; Unique Protocol ID: PPA-EF-SR-2025; PACTR secondary application/record ID: 41034
**Ethics approval**	CPP N 13/2025; Local Research Ethics Committee at the High Institute of Sports and Physical Education of Ksar-Said, University of Manouba, Tunisia; obtained before participant recruitment/intervention.
**Study design summary**	Two-arm, parallel-group, single-blind randomized controlled trial; preschool children; EG n=19, CG n=29; 8-week PPA intervention versus regular physical education; baseline and post-intervention assessments; study start 7 April 2025; completion/final follow-up 23 May 2025.

Checklist note: CONSORT is the primary reporting guideline because the study is reported as a randomized controlled trial. This revised checklist adds explicit ethics approval and transparent registration clarification for MDPI editorial assessment.

Section / Topic	Item	Descriptor	Reported?	Manuscript location	Author response / final action
Title and abstract	1a	Title identifies the study as a randomized trial.	Yes	Title/Abstract	The manuscript identifies the work as an exploratory randomized controlled trial.
Title and abstract	1b	Structured summary of design, methods, results, and conclusions.	Yes	Abstract, pp. 1-2	The abstract reports design, groups, intervention, outcomes, statistical approach, main results, and cautious conclusion.
Open science	2	Registry name, identifier, URL, and registration date.	Yes - disclosed as retrospective	Abstract; Methods 2.1; trial registration statement	ClinicalTrials.gov NCT07678294, Unique Protocol ID PPA-EF-SR-2025, public release 25 June 2026, and PACTR secondary application/record ID 41034 are disclosed. The retrospective nature of ClinicalTrials.gov registration is explicitly stated.
Open science	3	Protocol and statistical analysis plan access.	Partial / transparent	Methods; registry records; supplementary clarification	No separate public SAP is identified. The approved study procedures are described in the manuscript and registry records; the registration delay is explained transparently.
Open science	4	Data, code, and materials availability.	Yes / restricted	Data Availability Statement	De-identified child participant data should remain available only on reasonable request and subject to ethics/data protection requirements.
Funding and COI	5a	Funding/support and role of funder.	Yes	Funding statement	State funding/support and clarify whether the funder had no role in the study design, data collection, analysis, interpretation, or publication decision.
Funding and COI	5b	Financial and other conflicts of interest.	Yes	Conflicts of Interest	Declare no conflicts or list relevant financial/non-financial interests.
Introduction	6	Scientific background and rationale.	Yes	Introduction, pp. 2-3	The cognitive-motor/playful physical activity rationale and school-readiness context are reported.
Introduction	7	Specific objectives related to benefits and harms.	Yes / clarified	End of Introduction; Methods/Results	Aim and hypotheses are reported. Safety/adverse events should be explicitly summarized in Results, even if no events occurred.
Methods	8	Patient/public involvement in design, conduct, or reporting.	N/A / disclosed	Checklist note or Methods	No patient/public involvement is reported for protocol development; not applicable for this preschool intervention unless otherwise documented.
Methods	9	Trial design, allocation ratio, framework, and unit of randomization.	Yes	Methods 2.2, pp. 5-6	Single-blind, two-arm, parallel-group RCT; individual child-level randomization; EG n=19 and CG n=29 due to simple randomization.
Methods	10	Important protocol changes after commencement, with reasons.	Yes / transparent	Methods; Limitations; registration clarification	No intervention, outcome, allocation, or analysis change is attributed to registration timing. Retrospective

					registration is disclosed separately.
Methods	11	Trial settings and locations.	Yes	Methods 2.1, p. 4	Local preschools in Tunisia/Ksar-Said-Manouba context are reported.
Methods	12a	Eligibility criteria for participants.	Yes	Methods 2.1, p. 4	Inclusion, exclusion, and withdrawal criteria are reported; age range aligns with registry wording (55-64 months).
Methods	12b	Eligibility criteria for sites and intervention providers, if applicable.	Yes / partially applicable	Methods 2.3	Preschool setting and instructor/training standardization are reported; detailed site eligibility is not applicable beyond participating preschool settings.
Methods	13	Intervention and comparator details sufficient for replication.	Yes	Methods 2.3, pp. 6-7	PPA content, cognitive challenges, group delivery, duration, frequency, instructor, and conventional PE comparator are described.
Methods	14	Pre-specified primary and secondary outcomes, variables, metrics, aggregation, and time points.	Yes	Methods 2.4; registry outcome section	Primary focus: school readiness. Secondary outcomes: executive-function measures. Baseline and post-intervention assessment time points are described.
Methods	15	How harms/adverse events were defined and assessed.	Clarified	Methods/Results; checklist action	Because this was a supervised school-based physical activity intervention, adverse/unintended events should be summarized directly in Results. If none occurred, state that no serious adverse events, intervention-related injuries, or discontinuations were reported.
Methods	16a	Sample size determination and assumptions.	Yes	Methods 2.1 or statistical analysis, p. 4	G*Power, alpha, power, effect size, target sample, and enrolled sample are reported.
Methods	16b	Interim analyses and stopping guidelines.	N/A / clarified	Methods/Results	No interim analyses or formal stopping rules were planned for this short educational/behavioral intervention; the trial ended as planned after 8 weeks.
Methods	17a	Who generated random allocation sequence and method used.	Yes	Methods 2.2, p. 5	Independent researcher; computer-generated simple randomization.
Methods	17b	Type of randomization and restrictions.	Yes	Methods 2.1-2.2, pp. 4-5	Simple randomization without blocking or stratification; unequal group sizes are disclosed.
Methods	18	Allocation concealment mechanism until assignment.	Yes / clarified	Methods 2.2; registry	Allocation concealment from outcome assessors until baseline testing completion is reported; use the most accurate mechanism wording in the final manuscript.
Methods	19	Implementation: who enrolled, who assigned, and access to sequence.	Yes / clarified	Methods 2.2	Randomization sequence generation and assessor independence are described. Keep final manuscript wording explicit about enrolment and assignment roles.
Methods	20a	Who was blinded after assignment.	Yes	Methods 2.2, p. 5	Outcome assessors were blinded; participants, parents, teachers, and intervention providers were not blinded due to intervention nature.
Methods	20b	How blinding was achieved and similarity of interventions, if blinded.	Yes / clarified	Methods 2.2	Assessor blinding and non-feasibility of participant/provider blinding are explained.
Methods	21a	Statistical methods for primary/secondary outcomes and harms.	Yes	Statistical Analysis	Mixed ANOVA, Hedges g, ANCOVA sensitivity/baseline adjustment, alpha, and software should be reported.
Methods	21b	Definition of analysis population and analysis group.	Yes	Methods/Results participant flow	All 48 randomized children completed post-test and were retained; analysis as allocated.
Methods	21c	Handling of missing data.	Yes	Statistical Analysis; Results	No post-randomization losses; if no outcome values were missing, state this directly.
Methods	21d	Additional/subgroup/sensitivity analyses, pre-specified or post hoc.	Yes	Statistical Analysis; Results	ANCOVA sensitivity analyses are reported and interpreted cautiously.
Results	22a	Participant flow numbers for assignment, intervention received, and primary-outcome analysis.	Yes	CONSORT flow diagram; Methods 2.2	Flow diagram shows n=48 randomized, EG n=19, CG n=29, no losses, all analyzed.

Results	22b	Losses and exclusions after randomization, with reasons.	Yes	CONSORT flow diagram; Methods 2.2	No withdrawals, no losses to follow-up, no exclusions from analysis.
Results	23a	Recruitment and follow-up dates.	Yes	Methods 2.2; trial registration statement	Actual study start 7 April 2025; final follow-up/completion 23 May 2025.
Results	23b	Why trial ended or stopped, if relevant.	N/A / clarified	Methods/Results	The intervention ended as planned after the scheduled 8-week period; no early stopping reported.
Results	24a	Intervention/comparator actually administered, adherence/fidelity.	Yes / limitation disclosed	Methods 2.3; Limitations	Program duration/frequency are reported. Missing participant-level attendance, HR/RPE intensity, and quantitative fidelity data remain explicit limitations.
Results	24b	Concomitant care received during trial for each group.	Yes / clarified	Methods/Results	Children received assigned PPA or regular PE; no additional structured cognitive-motor intervention should be stated if applicable.
Results	25	Baseline demographic and clinical characteristics by group.	Yes	Baseline table; Results	Group baseline demographics and outcome values are reported; baseline imbalance is addressed.
Results	26	Numbers analysed, results for each outcome, effect sizes, and precision.	Yes	Results tables	n per group, mean/SD, Group x Time effects, partial eta squared, Hedges g, and adjusted sensitivity analyses are reported.
Results	27	All harms or unintended events in each group.	Clarified / add direct statement	Results	Add/retain a concise statement on adverse events or unintended intervention-related events for EG and CG.
Results	28	Ancillary analyses, subgroup/sensitivity analyses, pre-specified vs post hoc.	Yes	Results; response revisions	ANCOVA sensitivity analyses and cautious interpretation are reported.
Discussion	29	Interpretation consistent with results, balancing benefits and harms and considering evidence.	Yes	Discussion; Conclusion	Interpretation is cautious/preliminary; BVMT-R overinterpretation is avoided.
Discussion	30	Trial limitations: bias, imprecision, generalizability, multiplicity.	Yes	Limitations paragraph	Small sample, unequal groups, baseline imbalance, retrospective registration, non-blinded teacher ratings, missing fidelity/intensity/rater-reliability data are disclosed.

Source guideline: Prepared using the CONSORT 2025 reporting guideline structure and official CONSORT resources. CONSORT is the primary reporting guideline for this randomized controlled trial.
